# Bidirectional Relationship Between Language Ability and Internalising/Externalising Behaviour from Early to Late Childhood: Findings from a Chilean Cohort

**DOI:** 10.1007/s10802-025-01302-4

**Published:** 2025-03-10

**Authors:** Ricardo Mellado

**Affiliations:** Social Research Institute, University College of London, 55 - 59 Gordon Square, London, WC1H 0NU UK

**Keywords:** Children, Internalising behaviour, Externalising behaviour, Language ability, Cross-lagged, Child development

## Abstract

Language skills, along with emotional (internalising) and behavioural (externalising) problems, are known to independently influence long-term outcomes. However, the bidirectional relationship between these factors over time, particularly during childhood and early adolescence, remains underexplored. This study used a sample of 3,772 children from the Chilean Longitudinal Survey of Early Childhood to assess bidirectional effects between these domains at ages 2–4, 4–6, and 10–12. Externalising and internalising problems were assessed using the Child Behaviour Checklist, and language ability was measured with the Peabody Picture Vocabulary Test. Structural Equation Modelling, specifically the Random Intercept Cross-Lagged Model, revealed: (a) bidirectional effects between externalising behaviour and language skills, with stronger effects of externalising behaviour on language; (b) externalising symptoms predicted increased internalising symptoms across all periods; (c) internalising symptoms at ages 4–6 predicted lower levels of externalising behaviour at ages 10–12, suggesting a protective role; and (d) gender-specific patterns, with males showing bidirectional effects between externalising symptoms and receptive language, while females showed stronger unidirectional effects of early externalising behaviour on later internalising behaviour. These findings highlight the importance of early monitoring of externalising symptoms and language challenges to mitigate future issues.

**Supplementary Information:**

The online version contains supplementary material available at 10.1007/s10802-025-01302-4.

Early language skills and social-emotional development form essential building blocks for a child’s future success in life. Poor language skills and increased emotional and behavioural challenges in childhood are each independently linked to negative outcomes later in life, such as poverty, mental health disorders, and social exclusion (Johnson et al., 2010; Dekker et al., 2007; Feinstein & Bynner, 2004; Schoon et al., 2015; Fergusson et al., 2005). However, less is known about how these domains of development are interrelated throughout childhood, particularly within population samples measured at multiple time points. Poorer language skills may act as a risk factor for the development of internalising and externalising behaviours, and these behaviours, in turn, may serve as risk factors for each other and for language development. This study examines how language skills (i.e., receptive language), behavioural challenges (i.e., externalising behaviour), and emotional symptoms (i.e., internalising behaviour) influence each other longitudinally across three time points in a population-based sample of Chilean children. By identifying unidirectional or bidirectional effects between these domains, this research aims to provide insights into the optimal timing, targets, and strategies for early interventions. Strengthening cognitive, emotional, and behavioural functioning in children during early developmental stages has the potential to prevent the escalation of language difficulties and behavioural and emotional challenges over time (Masten & Cicchetti, 2010).

## Language, Internalising and Externalising Behaviour During Childhood

The terms “internalising” and “externalising” denote children’s behavioural and emotional issues, respectively. Externalising behaviour problems encompass a range of outwardly directed behaviours that negatively impact a child’s interactions with their environment, including disruptive, hyperactive, and aggressive behaviours (Campbell et al., 2000; Eisenberg et al., 2001). Internalising behaviour refers to issues affecting the child’s internal psychological state, such as withdrawal, anxiety, inhibition, and depression, reflecting turmoil within the individual’s internal world (Campbell et al., 2000). Language skills in children encompass the development and use of communication abilities, including understanding and producing spoken words, as well as non-verbal cues, to effectively share thoughts, express needs, and interact with others (Hoff & Hoff, 2009). These developmental domains appear early in life and tend to exhibit higher levels of stability across childhood. For example, a study of a language community cohort (McKean et al., 2017) found that 94% of children aged 4 to 11 with adequate language abilities maintained their skills over time, whereas those with low language skills tended to remain low during that period. Similarly, internalising and externalising behaviours show significant stability from early childhood through pre-adolescence. Caspi et al. (1996) observed that both types of behaviours maintain a consistent pattern from ages 2–3 to 10–11 years, with externalising behaviour exhibiting slightly greater stability.

During childhood, internalising and externalising symptoms tend to co-occur. Possible reasons for this comorbidity include shared underlying causal factors, such as higher-order dimensions of personality or temperament (e.g., negative emotionality or low conscientiousness; Weiss et al., 1998), common genetic liability (O’Connor et al., 1998), or a shared environment that fosters maladaptive responses (Caron & Rutter, 1991; Gilliom & Shaw, 2004; Lilienfeld, 2003; Reitz et al., 2005). In addition, deficits in emotion regulation are often implicated as a key factor influencing both domains, as difficulties in managing emotional responses can increase susceptibility to both anxiety and depression (internalising) and impulsivity or aggression (externalising; Cole & Hall, 2008). Similarly, impairments in executive functioning, including poor inhibitory control and working memory, have been shown to underlie both the rigid, perseverative thinking linked to internalising behaviours and the impulsive, risk-taking tendencies characteristic of externalising behaviours (Yang et al., 2022).

Additionally, research suggests that the co-occurrence between externalising and internalising behaviours is partly due to the longitudinal relationship between these domains. Numerous studies consistently show that early externalising behaviour influences the later development of internalising symptoms (Boylan et al., 2010; Flouri et al., 2019; Herrenkohl et al., 2010). This relationship aligns with the “failure theory,” which posits that defiant behaviours, such as aggression or rule-breaking, can lead to social isolation and rejection by peers. Consequently, social isolation increases the risk of developing internalising symptoms, such as anxiety and depression (Capaldi, 1991). While some evidence supports unidirectional effects from internalising to later externalising behaviour in community samples (Van der Ende et al., 2016) and population samples (Flouri et al., 2019), this effect is less consistent and weaker compared to the reverse relationship. Moreover, as suggested by some previous literature, higher levels of internalising behaviour could reduce later externalising symptoms (Boylan et al., 2010; Weeks et al., 2016). This is supported by the idea that more internally focused youths are less likely to engage with deviant peers or take high-risk behaviours (Mesman & Koot, 2001), especially during the transition from childhood to early adolescence (Masten & Cicchetti, 2010).

Language ability tends to co-occur with both internalising and externalising behaviours during childhood (Hentges et al., 2021), as evidenced by studies exploring these relationships concurrently (Hughes et al., 2016) and longitudinally (Girard et al., 2016; Morgan et al., 2015). Several theoretical explanations have been proposed for this association. Firstly, common risk factors related to neurodevelopmental problems (e.g., delays in basic attentional, perceptual, and motor functions), maternal education, and socioeconomic factors may contribute to simultaneous problems in behavioural and emotional adjustment as well as lower language skills (Moffitt, 1990). Secondly, given that language acts as a regulatory mechanism for self-control (e.g., private speech), children with lower language skills may find it challenging to manage their emotions under stress. This difficulty can lead to frustration, which may manifest as depression and withdrawal and/or as aggressive and disruptive behaviours. Thirdly, limited language skills may reduce a child’s ability to reason or express themselves verbally, thereby hindering social interactions. Specifically, inadequate language skills can lead to misunderstandings and misinterpretations, increasing the likelihood of conflict and aggressive responses among peers and within the family, thus intensifying externalising behaviours (Cole et al., 2010). Furthermore, limited language proficiency may promote social withdrawal and a lower self-concept, which could exacerbate internalising behaviours. Fourthly, early manifestations of internalising symptoms such as anxiety and depression, as well as externalising behaviours like aggression and defiance, may negatively influence language development by reducing the frequency and quality of interactions, thereby restricting exposure to rich linguistic input (Carpenter & Drabick, 2011; Ford et al., 2018).

When the longitudinal relationships among language skills, externalising behaviour, and internalising behaviour have been examined, the evidence has been mixed. A study tracking 224 children (ages 4–14) from a community sample found that weaker language skills predicted higher internalising behaviour over time, but neither externalising nor internalising behaviours predicted later language difficulties (Bornstein et al., 2013). Another community sample study, consisting of 585 children aged 7–13, found bidirectional relationships between these variables, with language skills being a stronger predictor of later emotional and behavioural problems than the reverse (Petersen et al., 2013). By contrast, data from the large-scale Millennium Cohort Study (*n = *10,876) revealed consistent associations between early externalising behaviour and later language skills and internalising behaviour throughout childhood, while language skills predicted these behaviours only during late childhood (ages 7–11) (Tamayo et al., 2024). In summary, the available evidence suggests that children’s internalising symptoms, externalising symptoms, and language development generally (1) emerge early in childhood and demonstrate stability over time, and (2) tend to co-occur. Additionally, (3) there is evidence indicating that problems in one domain may lead to difficulties in other developmental areas, as well as evidence of simultaneous bidirectional effects between language skills and both internalising and externalising behaviour. However, studies vary in the strength and consistency of these effects across different periods of childhood. Despite these findings, ongoing research faces three significant limitations. Firstly, most studies assessing unidirectional or bidirectional effects between these domains rely on clinical or community samples with small sample sizes (Bornstein et al., 2013; Van der Ende et al., 2016). Small sample sizes pose challenges for generalisability, as they may disproportionately reflect the unique characteristics of the community being studied. This can skew reported associations, leading to inflated estimates—since small samples are more sensitive to outliers and extreme values—or occasionally deflated estimates if key subgroups or traits are underrepresented. Such biases are particularly likely if the sample fails to capture the full range of developmental trajectories. Secondly, some of these studies cover short periods of childhood (Bornstein et al., 2013; Girard et al., 2016) or begin in later stages of development (Petersen et al., 2013; Van der Ende et al., 2016), limiting the depth of temporal analysis. Shorter timeframes may miss cumulative effects, while later study starts overlook critical early interactions that shape developmental trajectories. Thirdly, most studies measure the same construct across waves using different assessment tools (Flouri et al., 2019; Masten et al., 2005; Tamayo et al., 2024; Weeks et al., 2016), which undermines consistency and comparability over time, potentially resulting in biased estimates. Finally, while recent studies using population samples (e.g., Flouri et al., 2019; Tamayo et al., 2024) represent significant progress in incorporating a broader range of relevant covariates compared with earlier studies based on smaller community samples, some key variables—such as maternal cognitive skills and executive functions, which are potential confounders in the relationships between internalising behaviour, externalising behaviour, and language skills—remain underrepresented.

Beyond these methodological challenges, there is a growing recognition that much of the existing research has been conducted within high-income, Western contexts, particularly in Europe and North America. As a result, findings from these settings may not be directly applicable to other regions with distinct socio-cultural and economic environments. Chile, as a South American country with notable socio-economic disparities and a unique cultural context, provides an essential opportunity to investigate these relationships using established research methodologies that have been successfully applied in other countries. Despite significant economic improvements over the past decades, Chile remains one of the most unequal countries within the OECD, with a large proportion of children facing developmental delays and socio-emotional challenges (Morales et al., 2024). Furthermore, cultural factors such as Chile’s collectivist values emphasizing hierarchy and obedience may influence the way children experience and express internalising and externalising behaviours. Research indicates that authoritarian parenting, prevalent in Chile, is a significant risk factor for both internalising and externalising problems, with studies of Chilean preschoolers showing a clear link between such parenting styles and increased behavioural and emotional difficulties (Bedregal et al., 2023).

This research situates the study within the Chilean context, building on existing knowledge of bidirectional relationships between language skills and socio-emotional development, while offering new insights specific to this socio-economically diverse population. The findings could have significant implications for interventions aimed at addressing developmental challenges faced by socioeconomically disadvantaged children in Chile. Furthermore, the results may contribute to national policy initiatives, such as the Chile Crece Contigo program, which is a government-driven effort to promote child development from conception to preschool. By examining the interplay between language development and socio-emotional challenges, this study aspires to provide actionable insights that can enhance these interventions and help ensure that children from disadvantaged backgrounds receive the support they need to thrive.

## Overview of this Study

This study contributes to the existing literature by examining the longitudinal bidirectional relationships between language skills and socio-emotional development—specifically internalising and externalising behaviours—spanning early childhood to puberty. Drawing on a population-based sample of Chilean children aged 2–4 to 10–12, language development is assessed using the Spanish version of the Peabody Picture Vocabulary Test (PPVT), which measures receptive language abilities. Socio-emotional development is evaluated using the Child Behaviour Checklist (CBCL), providing insights into internalising and externalising behavioural symptoms.

To examine these relationships, Random Intercept Cross-Lagged Panel Models (RI-CLPM) were employed across three waves of data. The RI-CLPM extends traditional cross-lagged panel models by incorporating random intercepts for repeatedly measured outcomes, thereby separating within-person variance (temporal deviations from an individual’s expected scores) from between-person variance (stable differences across individuals). Recently adopted in studies such as Flouri et al. (2019) and Tamayo et al. (2024), this approach captures “pure” longitudinal changes, unconfounded by stable individual differences or between-person variability. Functioning effectively as a multilevel model, the RI-CLPM provides a robust framework for analysing developmental cascades—dynamic, bidirectional influences—among language skills, internalising behaviour, and externalising behaviour over time (Hamaker et al., 2015).

**Fig. 1:**
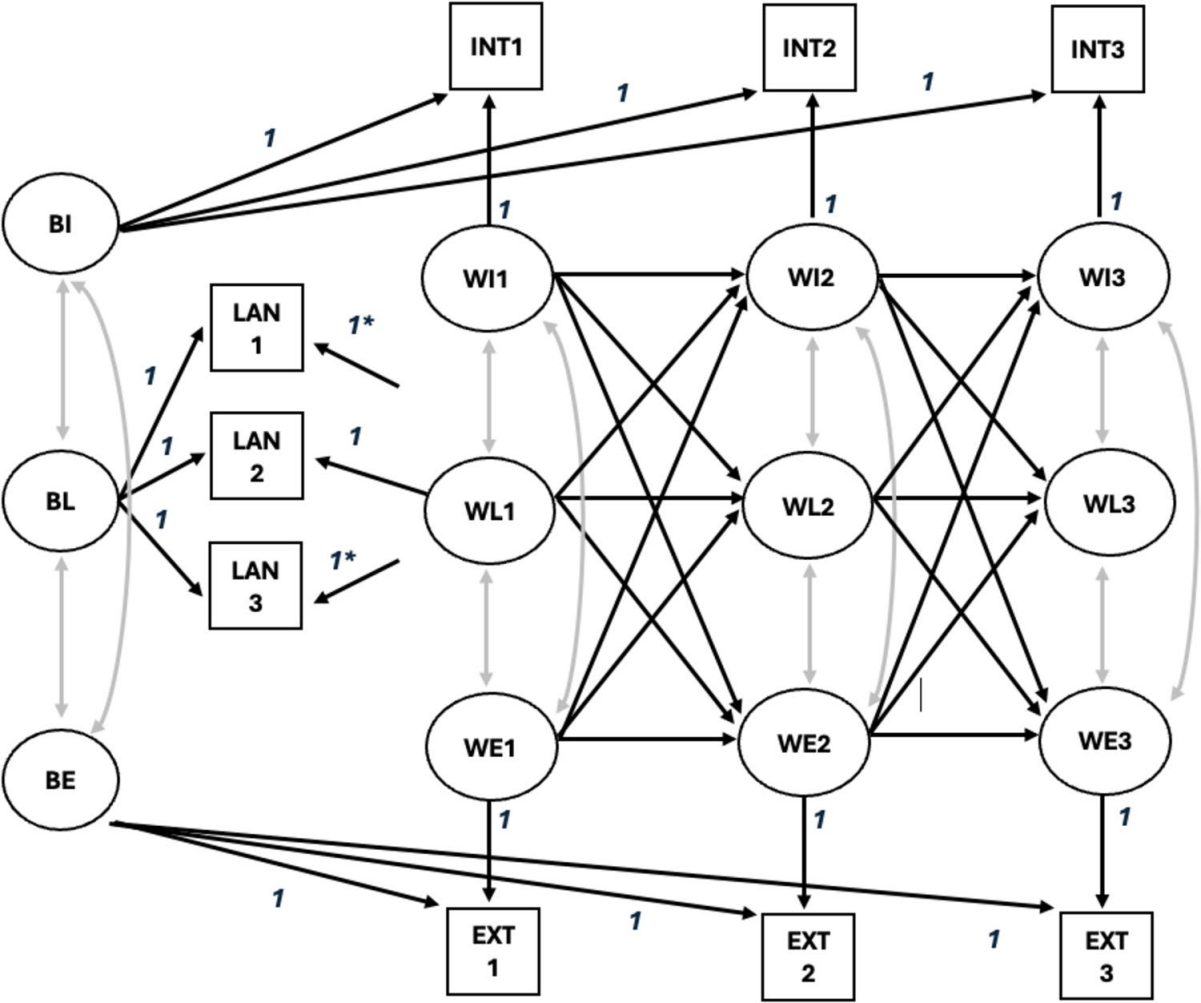
The diagram presents a three-wave Random Intercept Cross-Lagged Panel Model (RI-CLPM) illustrating the relationships between Externalising Behaviour (WE), Internalising Behaviour (WI), and Language Skills (WL). The curved arrows represent covariances and residual covariances. Arrows marked with an asterisk “*” should connect to their respective latent variables, as demonstrated with “WL1,” but are purposefully omitted in the diagram for simplicity

The conceptual RI-CLPM model is depicted in Fig. 1. Random intercepts at the between-person level capture the time-invariant, trait-like stability of language skills (BL), internalising behaviour (BI), and externalising behaviour (BE). Latent time-specific factors represent within-person changes around each individual’s expected score in these outcomes across the three waves (WL, WI, and WE, respectively). Autoregressive and cross-lagged paths were estimated between the within-person effects of children’s language skills, internalising behaviour, and externalising behaviour. Within-time correlations among these domains were also allowed to covary at each wave, controlling for time-specific confounders that might simultaneously impact them and thereby serving as controls in the cross-lagged analysis. 

Based on prior research, the anticipated outcomes of this study include significant comorbidity among internalising behaviour, externalising behaviour, and language skills, with all three domains expected to covary across the three time periods. Each domain is also anticipated to exhibit stability over time, independent of the covariance with other domains at each wave. Regarding bidirectional effects, early externalising behaviour is expected to predict later internalising problems, while the reverse relationship (from internalising to externalising behaviour) is likely to be weaker, less consistent, or even negative. Bidirectional effects between externalising behaviour and language skills are anticipated, while significant bidirectional effects between internalising behaviour and language skills are not expected, based on limited evidence from previous studies (e.g., Petersen et al., 2013).

## Method

### Sample

The data for this study were obtained from the Chilean Longitudinal Survey of Early Childhood (ELPI), a nationally representative survey. ELPI conducts face-to-face interviews and collects two types of data: a socio-demographic survey administered to all mothers and a battery of tests assessing cognitive, socio-emotional, and anthropometric development in children and their mothers. The 2010 wave included a sample of approximately 15,000 children aged 6 months to 5 years, randomly selected from official administrative birth records of children born between January 2006 and August 2009. In 2012, a second wave targeted the same sample as in 2010, along with an additional 3,000 children born between September 2009 and December 2011, aged 6 months to 7 years. The third wave, conducted in 2017, included participants from the previous waves and added approximately 5,000 children born between January 2012 and December 2016, ranging in age from 6 months to 12 years. The sample encompasses multiple cohorts of children, distinguished by their year of birth.

The ELPI survey includes various cognitive and socio-emotional tests. In this study, data from all three rounds of the survey were used to examine three outcomes. Language skills were assessed with the Peabody Picture Vocabulary Test (PPVT), which evaluates receptive vocabulary, vocabulary acquisition, and verbal intelligence in children over 30 months of age. Socio-emotional outcomes were examined using the Child Behavior Checklist (CBCL), which identifies various behavioural and emotional issues in children and adolescents over 18 months of age.

The longitudinal initial sample at wave 1 of the study consists of 7,022 children. Of this group, 3,514 are male (49.7%) and 3,557 are female (50.3%), with sex assigned at birth used as the classification criteria. The children are distributed across ages 2 (26.7%), 3 (50.8%), and 4 (22.5%), with 66% attending pres-chool. The mean age of their mothers is 30.2 years old (range: 15—54), and they have an average of 11.2 years of education (range: 1—20). Additionally, 31% of these households are composed of children living without their biological father.

The inclusion criteria for this research consider children aged 30 to 58 months at the initial assessment in 2010. This specific age range is chosen because the application of the PPVT assessment in ELPI is designed for children aged 30 months and above. The exclusion criteria for the follow-up sample include cases where the child was not living with their biological mother at the first wave, as well as incomplete data in the first wave concerning child behavioural assessment, language skills scores, and other covariates considered in the model.

The final sample consisted of 3,772 children who participated in all three waves of the study, while 3,250 individuals were excluded due to attrition. Table 1 compares the demographic characteristics of the included and excluded samples, revealing significant differences in maternal and household attributes. Specifically, the longitudinal sample overrepresented mothers with lower cognitive abilities (measured by WAIS scores) and households with lower wealth. To address these disparities and enhance the generalizability of the findings, sampling weights were applied to the cross-lagged estimates. These weights, calculated based on the probability of sample selection, adjusted the sample distribution to better reflect the population’s characteristics (Behrman et al., 2010).

**Table 1:** Mean and proportion comparison between excluded and included sample

	Sample included (*n = *3,772)	Sample excluded (*n = *3,250)	*p*-value
Gender (1 = female)	0.50	0.51	0.65
Biological Father present (1 = yes)	0.30	0.32	0.02
WAIS vocabulary	8.02	8.33	0.00
WAIS numerical	6.85	7.10	0.00
Mother age (first wave)	30.66	29.62	0.00
Postpartum depression (1 = no)	0.90	0.89	0.15
Preterm birth (1 = no)	0.90	0.89	1.00
Household Wealth Quintiles	3.02	3.18	0.00
Caregiver does not yell (1 = yes)	0.79	0.78	0.34
Caregiver shows affection (1 = yes)	0.79	0.78	0.24
Backward Digit Span Task	45.49	45.72	0.10

Differences between the two samples were assessed using independent sample t-tests for continuous variables and chi-squared tests for categorical variables

### Ethical Approval

Ethical evaluation of the study was conducted by the Microdata Center of the University of Chile. Informed consent was obtained from the parents of all children included in the study.

## Measures

### Peabody Picture Vocabulary Test

The Spanish version of the Peabody Picture Vocabulary Test-III (PPVT-III) is a standardized assessment tool designed to measure receptive vocabulary, evaluating an individual’s ability to understand and comprehend words and vocabulary (Dunn & Dunn, 1986). The PPVT-III has demonstrated very high internal consistency, with a Kuder-Richardson Formula 20 (K-R 20) coefficient of 0.98 (Dunn & Dunn, 1986), indicating excellent reliability. In addition, the PPVT-III has been validated for use across diverse Spanish-speaking communities, including adaptation for Chilean language norms (e.g., replacing less frequent words with more frequent terms) to ensure cultural relevance (Strasser et al., 2010). The test has also shown a strong correlation (*r = *0.95) between different versions of the assessment, further supporting its cross-version reliability, as reported by Strasser et al. (2010). This strong reliability and validity ensure that the PPVT-III provides an accurate measure of receptive vocabulary across different contexts.

During the PPVT-III, children are presented with a series of pictures and asked to select the picture that corresponds to the word spoken by the examiner from a set of four multiple-choice options. The test assesses the individual’s understanding of spoken language and their ability to match visual stimuli with appropriate vocabulary words. Each correct response is assigned a score of 1, while incorrect responses receive a score of 0. The test continues until the child provides six consecutive incorrect answers. The examiner records the test-taker’s responses to calculate a raw score. The PPVT-III has been standardized to establish age-based norms, which are used to convert the raw score into a standard score or percentile rank.

### Child Behaviour Checklist

The Child Behaviour Checklist (CBCL) is a widely used tool for assessing the behavioural and emotional functioning of children. It includes two versions: a preschool version for children aged 1.5 to 5 years and a school-age version for children aged 6 to 18 years. The CBCL categorizes behaviours into two higher-order factors: internalising and externalising behaviour, derived from subscales that capture distinct aspects of behavioural and emotional functioning (Achenbach & Rescorla, 2001).

The CBCL questionnaire, completed by parents, consists of 99 items in the preschool version and 112 items in the school-age version. Each item is rated on a scale of 0 (not true), 1 (somewhat or sometimes true), or 2 (often true). For the preschool version, a subset of 60 items is used: 36 for internalising behaviour and 24 for externalising behaviour. In the school-age version, a subset of 51 items is used: 26 for internalising behaviour and 25 for externalising behaviour. Raw scores are standardized to a 28–100 scale, with higher values indicating greater frequency or intensity of behaviours. Standardization compares an individual’s scores to a reference group of children of similar age and gender. These scores are converted into T-scores, with a mean of 50 and a standard deviation of 10, allowing comparisons to average behaviours within the reference group.

To measure emotional and behavioural characteristics of children, the CBCL uses age-specific subscales for internalising and externalising behaviour. For children aged 1.5 to 5 years, internalising behaviour is measured through the following subscales: emotional reactivity (9 items; e.g., “gets upset with new people or situations”), anxious/depressed (8 items; e.g., “looks unhappy”), withdrawn (8 items; e.g., “shows little interest”), and somatic problems (11 items; e.g., “does not eat well”). In the school-age version (ages 6 to 18), internalising behaviour includes anxious/depressed (13 items; e.g., “talks about killing himself”), withdrawn (8 items; e.g., “is reserved”), and somatic problems (5 items; e.g., “has nightmares”).

Externalising behaviour in children aged 1.5 to 5 years includes the subscales attention problems (5 items; e.g., “poor coordination or clumsiness”) and aggressive behaviour (19 items; e.g., “destroys family belongings”). In the school-age version, it includes rule-breaking behaviour (17 items; e.g., “runs away from home”) and aggressive behaviour (18 items; e.g., “threatens others”). (See Table S1 in the Supplementary Materials for CFA fit indices, including CFI, SRMR, and RMSEA, for internalising and externalising behaviour assessments.)

## Covariates

Children’s language ability, internalising symptoms, and externalising symptoms may be correlated due to shared underlying factors. Environmental influences, such as socioeconomic status (Christensen et al., 2014; Flouri & Midouhas, 2017; Hair et al., 2015), low parental education (Noble et al., 2015; Ormel et al., 2015), and household structure (e.g., single-parent households), significantly shape behavioural, emotional, and cognitive outcomes. These effects occur both directly, through impacts on brain development, and indirectly, through parenting style and parental involvement in learning. Executive function—a set of cognitive skills used to manage thoughts, emotions, and actions—affects behavioural and emotional regulation, thereby influencing internalising and externalising problems (Diamond & Ling, 2016). Key components of executive function, such as working memory and cognitive flexibility, are crucial for language development. Working memory helps children track words and meanings, while cognitive flexibility enables them to adapt to new vocabulary and rules (Gooch et al., 2016). Finally, early biological issues related to preterm birth increase the risk of cognitive, emotional, and behavioural challenges, primarily due to neurodevelopmental vulnerabilities, such as delays in cortical thinning (Arpi & Ferrari, 2013; Largo et al., 1986).

In line with the above, a set of time-variant and time-invariant covariates was selected. Among these are household variables, including socioeconomic status, which is measured through household wealth quintiles. These quintiles are derived from a principal component analysis of items related to household assets, dwelling ownership, and the number of bathrooms and sleeping-only rooms reported in the ELPI survey. Based on this analysis, individuals are ranked and divided into quintiles. Additionally, the presence of the biological father in the household is considered as a covariate.

Maternal characteristics include two indicators of skills from the Wechsler Intelligence Test (numerical ability and vocabulary), postpartum depression, and age. Child characteristics include preterm birth status and executive cognitive skills, assessed through the Backward Digit Span Task for working memory and attention.

Additional covariates measuring the household environment were considered in the analysis. The Home Observation for Measurement of the Environment (HOME), developed by Caldwell and colleagues (Bradley & Caldwell, 1984), comprises a series of dichotomous questions aimed at assessing emotional support and cognitive stimulation in the home environment, planned events, and the family that surrounds the child. Each round of ELPI incorporated a different set of questions from HOME; therefore, we included only the two variables that are common across all three rounds: whether the caregiver shows affection during the interview (caresses, kisses, cuddles) and whether the caregiver refrains from scolding, derogating, or yelling at the child during the interview. Most covariates demonstrated the anticipated associations with all three outcomes across the assessment points. However, preterm birth was an exception, showing no significant association with any of the outcomes at waves 1, 2, or 3, and was therefore excluded from subsequent analyses (see Table S2 [Supplementary Materials] for the bivariate correlation results between internalising behaviour, externalising behaviour and language skills and all the covariates).

### Empirical Strategy

Structural equation modelling was fitted using lavaan package in R software (Rosseel, 2012) and Mplus 8.11 (Muthen & Muthen, 2017) to examine the relationship among internalising, externalising and language skills. Specifically, random intercept cross- lagged panel models are used to assess if across the waves there is unidirectional and bidirectional effects between internalising and externalising symptoms, along with receptive language ability.

To test the full cross-lagged model, a series of three nested models were considered. Model 1 allows stability paths to vary freely within domains across waves while keeping cross-lagged paths fixed. In Model 2, the cross-lagged effects from all outcomes are allowed to vary, but the stability coefficients remain fixed across all waves. These two models are then compared with the unrestricted model, which allows both stability and cross-lagged effects to vary freely. To determine the acceptable absolute fit of the models, we used the standardized root mean square residual (SRMR), the comparative fit index (CFI), and the root mean square error of approximation (RMSEA). For the fit to be considered acceptable, the SRMR must be less than 0.08, the CFI must be greater than 0.90, and the RMSEA must be below 0.08. Maximum likelihood estimation with robust standard errors was employed to manage the skewed distributions of internalising and externalising problem scores within the analytic sample. Missing data on outcome variables were handled using full information maximum likelihood (FIML), which estimates parameters based on all available data under the assumption that the data are missing at random (MAR).

After selecting the model that best fits the data, an analysis of cascade effects between internalising behaviour, externalising behaviour, and language ability is conducted. First, bidirectional effects between these domains are estimated, adjusting solely for stability and cross-sectional associations. Then, the model is re-estimated with covariates included to determine whether the cross-lagged paths remain significant after adjustment. A multi-group analysis is then implemented to explore potential gender-based differences in these effects, in line with prior research suggesting gender differences in developmental trajectories and their interplay (Henrichs et al., 2013). Finally, a sensitivity analysis will be conducted using the anxiety subscale (for internalising behaviour) and the aggressive behaviour subscale (for externalising behaviour) of the CBCL, as these are the only scales common across the CBCL1 and CBCL2 assessments. This step is intended to ensure consistency across waves, address potential bias introduced by differences in subscale composition when using broader internalising and externalising scales, and evaluate whether the results hold after adjusting the model.

## Results

### Descriptive Analysis

The mean scores for internalising behaviour, externalising behaviour, and receptive language skills were examined across genders and assessment periods. Males exhibited significantly higher externalising behaviour scores at ages 2–4 and 4–6, with no significant differences observed at age 10–12. Additionally, males demonstrated lower receptive language skills at ages 2–4 and 4–6 but outperformed females at age 10–12. For internalising behaviour, no significant gender differences were found at ages 2–5 and 4–6. However, males had significantly higher scores at age 10–12. (See Table S3 [Supplementary materials] for the gender comparison of internalising, externalising, and language skills across the three waves.)

A multiple-group structural equation modelling (SEM) analysis was conducted to examine whether the relationships between externalising behaviour, internalising behaviour, and language skills varied by gender across the study waves. A chi-square difference test was performed to compare the unconstrained model (which allows paths to vary by gender) with the constrained model (which fixes paths to be equal across genders). The results (χ^2^ difference = 50.20, *p*-value = 0.03) indicated that at least one path differed between genders, which justifies the use of a multi-group analysis.

At the bivariate level, significant within-domain and between-domain correlations were observed among internalising behaviour, externalising behaviour, and language skills across all waves. Internalising, externalising, and language scores were positively correlated within their respective domains across all waves (externalising: *r = *0.27—0.42, *p* < 0.001; internalising: *r = *0.26 – 0.33, *p* < 0.001; language: *r = *0.37—0.42, *p* < 0.001). Furthermore, these estimates revealed significant correlations between the domains in each wave. Externalising and internalising behaviour were positively associated at all waves, with correlation coefficients ranging from *r = *0.58 at ages 2–5 to *r = *0.66 at ages 9–11. Language skills scores were negatively related to both behaviours, although these correlations were relatively modest, ranging from *r = *−0.05 (internalising, ages 9–11) to *r = *−0.16 (externalising, ages 2–5) (see Table S4 [Supplementary materials] for the cross-sectional correlations of internalising, externalising, and language skills).

### Developmental Cascades: Evaluating Model Fit

In Model 1, where both autoregressive effects (i.e., stability paths) and cross-lagged effects were constrained to be equal, a good fit was observed (CFI = 0.988, SRMR* = *0.040, RMSEA = 0.044). Model 2 further improved the fit by fixing the autoregressive coefficients while allowing the cross-lagged coefficients to vary freely (Chi-Square diff = 52.567; CFI = 0.992, SRMR = 0.038, RMSEA = 0.052). Finally, the full random intercept cross lagged model, which allowed both stability paths and cross-lagged effects to vary freely, significantly improved the fit (Chi-Square diff = 109.17; CFI = 0.999, SRMR = 0.010, RMSEA = 0.028). Therefore, the unrestricted model, where all paths were freely estimated, was chosen for further interpretation.

### Results

The estimates of standardized path coefficients for the cross-lagged model in the entire longitudinal sample are presented in Table 2. The first set of estimates shows the autoregressive and cross-lagged effects of externalising and internalising behaviour, along with language skills, without controlling for any covariates. The second set of estimates presents the same path coefficients, but with covariates included. A consistent pattern of autoregressive continuity is observed for externalising behaviour, internalising behaviour, and receptive language scores across all waves, indicating stability in these constructs over time. Specifically, path coefficients suggest that, after adjusting for covariates, early levels of externalising behaviour (β = 0.16 − 0.29), internalising behaviour (β = 0.09), internalising behaviour (β = 0.11), and PPVT scores (β = 0.09 − 0.14) significantly predicted later levels of the same constructs. While these coefficients may appear smaller than expected, this is a result of using the RI-CLPM, which separates stable between-person variance from within-person changes. Unlike the traditional CLPM, where autoregressive effects combine within- and between-person variance to indicate rank-order stability, the RI-CLPM captures only within-person carryover. This refinement leads to smaller or non-significant coefficients but provides a more nuanced understanding of developmental processes (see Table S5 [Supplementary materials] for the CLPM regression estimates).

**Table 2:** Regression coefficients for all paths in the Random Intercept Cross-Lagged Panel Model, with and without adjustment for covariates

Path Description	Path coefficient (no covariates)	Path coefficient (covariates)
T1 Externalising → T2 Externalising	0.30***	0.29***
T2 Internalising → T2 Externalising	−0.02	−0.04
T1 PPVT → T2 Externalising	−0.08***	−0.05*
T1 Externalising → T2 Internalising	0.07**	0.06*
T1 Internalising → T2 Internalising	0.10***	0.09**
T1 PPVT → T2 Internalising	−0.04	−0.02
T1 Externalising → T2 PPVT	−0.13***	−0.12***
T1 Internalising → T2 PPVT	0.01	0.03
T1 PPVT → T2 PPVT	0.13***	0.09**
T2 Externalising → T3 Externalising	0.16***	0.16***
T2 Internalising → T3 Externalising	−0.06*	−0.06*
T3 PPVT → T3 Externalising	−0.05*	−0.05*
T2 Externalising → T3 Internalising	0.11**	0.11***
T2 Internalising → T3 Internalising	−0.02	−0.01
T2 PPVT → T3 Internalising	0.00*	−0.01
T2 Externalising → T3 PPVT	−0.15**	−0.13***
T2 Internalising → T3 PPVT	0.04	0.03
T2 PPVT → T3 PPVT	0.17***	0.14***

T1 = Wave 1; T2 = Wave 3; T3 = Wave 3; Internalising = Internalising Behaviour; Externalising = Externalising Behaviour; PPVT = Language Skills

Secondly, the examination of cross-lagged effects between behavioural and emotional symptoms reveals a bidirectional relationship. Externalising behaviour has a more pronounced and consistent impact on subsequent internalising symptoms compared to the reverse. Specifically, higher levels of externalising behaviour at ages 2–4 significantly predicted increased internalising symptoms at ages 4–6 (β = 0.06, CI [0.01—0.11]). This pattern continued, with externalising behaviour between ages 4 and 6 predicting increased internalising behaviour at ages 10 and 12 (β = 0.11, CI [0.04—0.16]). These cross-lagged effects remained significant after adjusting for covariates. Conversely, the influence of internalising behaviour on subsequent externalising behaviour was less consistent, with only one significant path (from age 4–6 to age 10–12) after accounting for covariates (β = −0.06, CI [−0.11—−0.01]).

Finally, the estimates of bidirectional effects between behavioural symptoms and receptive language skills reveal that higher early externalising symptoms are associated with lower receptive language scores later on, observed from ages 2–4 to 4–6 (β = −0.12, CI [−0.17, −0.09]) and from 4–6 to 10–12 (β = −0.13, CI [−0.19, −0.08]). This pattern extends consistently in the reverse direction, albeit with smaller effects: the cross-lagged analysis identifies significant longitudinal effects where higher early receptive language ability at ages 2–4 predicts lower externalising behaviour at ages 4–6 (β = −0.05, CI [−0.11, −0.02]) and from 4–6 to 10–12 (β = −0.05, CI [−0.10, −0.01]). Furthermore, no significant paths were found between internalising behaviour and receptive language skills (see Table S6 [Supplementary materials] for the RI-CLPM model regression estimates with their respective confidence intervals for the full sample).

**Fig. 2:**
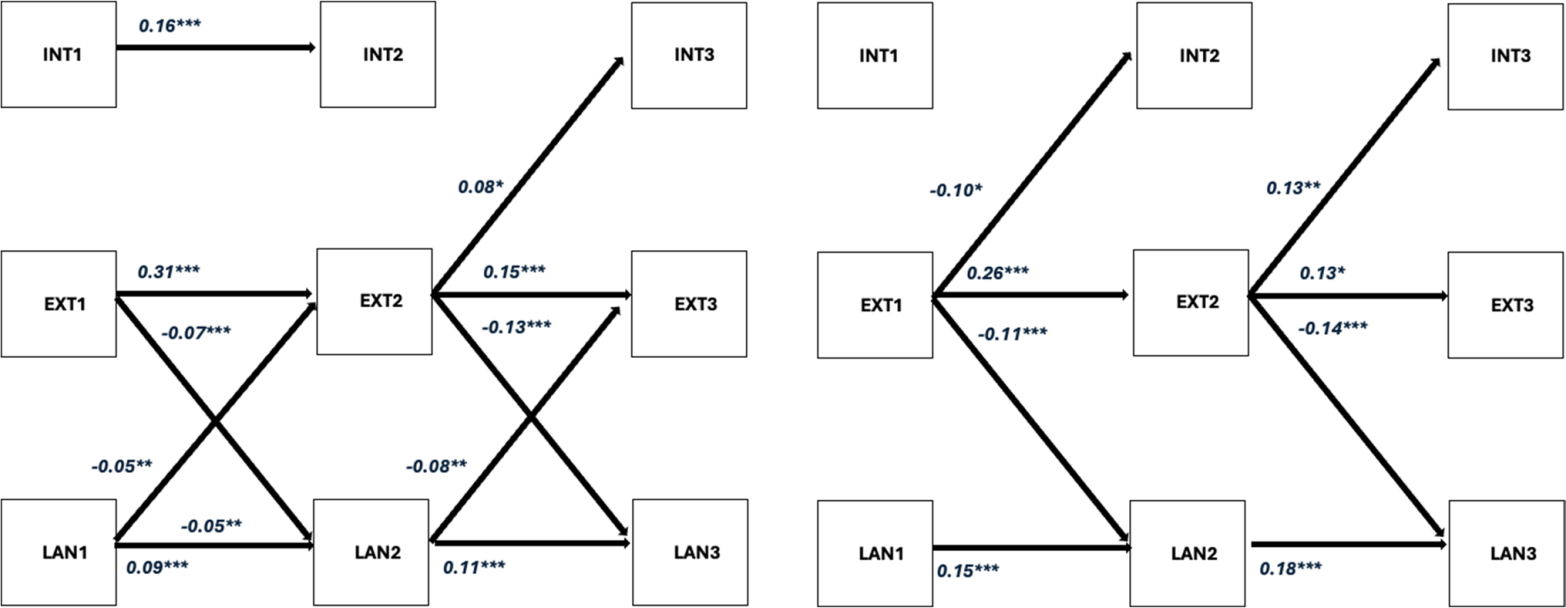
RI – CLPM for males (left) and females (right). Only significant standardized within effects are shown. Covariance across domains is omitted. INT = Internalising Behaviour; EXT = Externalising Behaviour; LAN* = *Language Skills Suffixes 1, 2 and 3 indicate wave 1, wave 2 and wave 3, respectively

The multigroup gender analysis, shown in Fig. 2, revealed distinct patterns. Consistent with the full sample analysis, early externalising behaviour predicted later internalising behaviour and language skills in both genders. However, for males, early externalising behaviour at ages 2–4 did not predict higher levels of internalising behaviour at ages 4–6, a relationship that was observed in females. The multigroup analysis further revealed that while the full-sample analysis indicated bidirectional effects between language skills and externalising behaviour across all waves, these effects held only for males. In females, externalising behaviour predicted language skills, but language skills did not significantly predict externalising behaviour. (See Tables S7 and S8 [Supplementary materials] for the RI-CLPM model regression estimates with their respective confidence intervals for females and males).

### Sensitivity Analysis

In the sensitivity analysis, the anxiety (for internalising behaviour) and aggression (for externalising behaviour) subscales of the CBCL were used, as they are consistent across both the CBCL1 and CBCL2 assessments. This approach produced results largely consistent with the original model, which included the broader internalising and externalising factors. However, some small differences were observed. In the adjusted RI-CLPM for the full sample, the autoregressive effects of externalising and internalising behaviour from T1 to T2 were stronger. Meanwhile, the impact of externalising behaviour in the cross-lagged effects was reduced, showing weaker associations with both internalising behaviour and receptive language skills at T1 and T2. Additionally, the cross-lagged effect between internalising behaviour and receptive language skills became significant from T2 to T3. Notably, these patterns were consistent across gender, indicating that the relationships observed were robust across both male and female subgroups. (See Tables S9, S10, and S11 (Supplementary materials) for regression estimates with their respective confidence intervals for the sensitivity analysis for the full sample, for males, and for females, respectively.)

## Discussion

A nationally representative, prospective cohort of children was used to examine bidirectional relationships between internalising symptoms, externalising symptoms, and language ability (i.e., receptive language) from ages 2–4 to 10–12 years across three waves. The findings provide the following insights: (a) difficulties in internalising behaviour, externalising behaviour, and language ability tend to emerge early, co-occur, and persist throughout childhood; (b) beyond the early onset, co-occurrence, and stability of emotional, behavioural, and language skills, the cross-lagged analysis identified consistent bidirectional effects between language skills and externalising behaviour; and (c) a less consistent relationship between externalising and internalising behaviour. These findings are discussed in greater detail below.

## Bidirectional Effects Between Internalising and Externalising Behaviour

Consistent effects were observed linking previous externalising behaviour to later internalising behaviour across all waves. The cross-lagged effect of externalising behaviour on internalising behaviour was found to be significant for both periods. Specifically, the effect from externalising behaviour at T1 to internalising behaviour at T2 was β = 0.06, and from externalising behaviour at T2 to internalising behaviour at T3 was β = 0.11. Both of these estimates align with the benchmark values for medium effects in the RI-CLPM (Orth et al., 2024). This finding supports the well-documented link between externalising and internalising symptoms observed in the literature (Gooren et al., 2011; Van Der Ende et al., 2016; Moilanen et al., 2010). For instance, one longitudinal study reported that six-year-old children with high externalising scores had a 40% probability of developing internalising symptoms by age nine (Willner et al., 2016). Further analysis by Gooren et al. (2011) explored the mechanisms underlying this association, revealing that peer rejection mediated the relationship between externalising symptoms and subsequent internalising symptoms in five-year-olds over a two-year span.

The reciprocal effect, where internalising symptoms predicted later externalising behaviour, was weaker, less consistent, and showed the opposite sign to the effect observed from externalising to internalising behaviour. Specifically, a negative relationship was observed from ages 4–6 to 10–12. The cross-lagged effect of internalising behaviour on externalising behaviour was small (β = −0.06), aligning with the benchmark for small effects in the RI-CLPM (Orth et al., 2024). This result suggests that internalising behaviour might actually lead to diminished levels of externalising behaviour. Previous research has hypothesized why this might occur: internalising behaviour could contribute to social withdrawal, thereby reducing opportunities for externalising behaviour. Alternatively, higher levels of internalising symptoms may encourage greater conscientiousness and caution, which could deter impulsivity and risk-taking behaviours (Masten et al., 2005). For example, one experimental study showed that inhibited 6-year-olds were less likely to cheat in tests, and similarly, inhibited 7-year-olds were less likely to adopt unfair strategies in competitive situations (Asendorpf & Nunner-Winkler, 1992). This pattern has also been observed in non-experimental settings. For instance, a population-based study of 6,425 Canadian children found that higher levels of internalising behaviour at ages 8–9 predicted lower externalising behaviour at ages 12–13 (Weeks et al., 2016).

The multi-group analysis highlighted subtle variations in gender-specific patterns. For females, significant pathways from externalising to internalising behaviour were observed consistently across all waves, spanning from ages 2–4 to 10–12. In contrast, for males, these significant pathways emerged only from ages 4–6 to 10–12. This finding aligns with previous population-based studies, such as Flouri et al. (2019), which identified a stronger and more consistent pattern of unidirectional effects from early externalising to late internalising behaviour in females compared to males. These gender-specific findings may reflect underlying developmental differences, as research suggests that girls are more likely to internalize emotional distress in response to externalising behaviour, whereas boys may express distress through additional externalising behaviour or alternative coping mechanisms (Masten et al., 2005).

## Bidirectional Effects Between Externalising Behaviour and Language Skills

Consistent bidirectional effects were observed between externalising behaviour and language skills, with externalising behaviour having a stronger impact on receptive language skills than the reverse. Specifically, higher early externalising symptoms were associated with lower language scores later on, with cross-lagged effects of β = −0.12 from ages 2–4 to 4–6 and β = −0.13 from 4–6 to 10–12, falling within the medium effect range (β = 0.07–0.12) according to the RI-CLPM benchmarks (Orth et al., 2024). In contrast, language ability also predicted later externalising behaviour, but with smaller effects: β = −0.05 from 2–4 to 4–6 and β = −0.05 from 4–6 to 10–12, which are within the small effect range (β = 0.03–0.07) in the RI-CLPM benchmarks (Orth et al., 2024). This finding aligns with studies conducted on similar population samples, such as the present study. For example, Tamayo et al. (2024) found that unidirectional effects of externalising behaviour on language skills were consistently stronger than the reverse across the entire period studied (ages 3 to 11). Similarly, Flouri et al. (2019) reported bidirectional effects between externalising behaviour and cognitive skills, with the effect of externalising behaviour on cognitive skills on cognitive development being stronger than the reverse.

Several theoretical mechanisms may explain the bidirectional relationship between externalising behaviour and language skills. First, it is argued that language allows children to communicate their needs effectively, and when language skills are limited, children may experience frustration and resort to disruptive behaviours like aggression to get their needs met (Keenan & Shaw, 1997). Second, language is essential for self-regulation; children with better language abilities may use language to guide their behaviour in challenging situations, while those with poorer language skills may struggle to control their emotions, which can lead to externalising behaviours (Vygotsky, 2012). Third, poor language skills may hinder social interactions, making children more likely to be rejected by peers, which in turn increases the likelihood of externalising behaviours (Menting et al., 2011). Moreover, externalising behaviours such as aggression may interfere with social interactions and reduce opportunities for verbal communication, further hindering language development (Lochman et al., 1989).

The multi-group analysis revealed a slightly different pattern: males exhibited consistent, unidirectional effects of receptive language skills on subsequent externalising behaviour, a trend not observed in females. This finding aligns with a recent meta-analysis by Hentges et al. (2021), which showed that child gender significantly moderates the relationship between language competence and externalising problems. Specifically, the association between language deficits and externalising behaviour strengthened as the percentage of boys in the sample increased. Koenen et al. (2006) suggested that this gender difference may be explained by the higher prevalence of early neurodevelopmental disorders in boys, such as low verbal IQ, as well as the fact that these disorders tend to exhibit a stronger correlation with externalising behaviour in boys compared to girls (Moffitt et al., 2001; Rutter, Caspi, & Moffitt, 2003).

## Strengths and Limitations

This study offers several strengths, including its large, population-based sample, a lengthy study period spanning key developmental transitions (from early childhood to puberty), and the consistent use of the same measurement instrument across waves to assess behavioural and language domains. Moreover, the study employs a novel statistical technique (RI-CLPM) to model within-individual longitudinal associations between internalising and externalising behaviours and receptive language skills, providing a robust foundation for causal inferences.

However, there are four key limitations to consider. First, internalising and externalising problems were reported solely by parents (primarily mothers), without triangulating data from other sources such as teachers or the children themselves. Second, while the interval between Waves 1 and 2 was relatively short (2 years), the gap between Waves 2 and 3 extended to 5 years, creating an inconsistency in time intervals that may introduce bias due to the uneven spacing between assessments. Third, given the correlational nature of the study and the presence of unmeasured common causes (e.g., genetic factors, peer rejection, and victimization) that could confound the relationships between these domains, causal inferences cannot be made. Peer rejection and victimization, in particular, are well-established risk factors for internalising and externalising behaviour, but reliable data on these variables were not collected in this study, partly due to the developmental stages of participants during the first two waves. Fourth, although the Peabody Picture Vocabulary Test was consistently used across all three waves to measure receptive language skills, future studies should investigate the bidirectional effects of behavioural and emotional symptoms alongside other language abilities, such as expressive language skills. Future research should aim to replicate and extend these findings over a longer time frame, control for additional covariates that may confound the cross-lagged analysis and examine different mediators to better understand how these developmental domains influence each other.

## Implications

The use of the Random Intercept Cross-Lagged Panel Model (RI-CLPM) to assess cross-lagged effects between different developmental domains in this study has significant methodological implications. These findings underscore the importance of employing statistical techniques that not only model how specific developmental domains evolve over time but also examine how these domains influence one another longitudinally. Furthermore, the application of RI-CLPM is methodologically valuable as it allows for the separation of between-person and within-person effects, offering a clearer understanding of how developmental changes unfold both across individuals and within the same individual over time.

Overall, the most consistent pattern observed is that externalising behaviour from ages 2–4 predicts later internalising behaviour and language skills through to ages 10–12. This effect remains significant in the full sample after controlling for all covariates. However, the gender-specific analysis highlighted nuanced differences: while early externalising behaviour predicted both internalising behaviour and language skills in males and females, the strength and consistency of these effects varied. For males, bidirectional effects between language skills and externalising behaviour were observed across all waves, whereas for females, language skills did not significantly predict externalising behaviour. Additionally, externalising behaviour consistently predicted later internalising behaviour in females, but this effect was less consistent in males. These findings emphasise the importance of considering gender-specific developmental trajectories when designing early interventions for externalising behaviour.

This research yielded an unexpected result: earlier internalising behaviour (ages 4–6) is linked to decreased externalising behaviour (ages 10–12). This finding adds to the growing evidence suggesting that traits related to internalising behaviour (such as behavioural inhibition) may serve as a protective factor against the development of deviant behaviour in certain situations and during specific developmental periods, particularly from later childhood into adolescence (Kerr et al., 1997). Ultimately, this result underscores the challenge of universally labelling behaviours as either solely good or bad, or risky or protective. Internalising symptoms are dynamic; they may serve protective roles under certain conditions while potentially increasing risks under others.

By addressing these issues early, it is possible to reduce the likelihood of future psychological maladjustment or compromised language abilities. Moreover, even modest improvements in health and well-being can lead to significant and meaningful benefits at the societal level, highlighting the tremendous value of early preventative measures. It is important to note, however, that these findings do not imply that every child who faces difficulties in one area will necessarily encounter challenges in others. Nevertheless, given the rapid development of externalising and internalising behaviours, as well as language skills, intervening early for language delays or behavioural issues could be both wise and cost-effective.

The findings suggest practical implications for governmental programs like *Chile Crece Contigo*, particularly in the context of its expanding Mental Health Support initiative. Evidence-based strategies such as social skills interventions—offering direct instruction in skills like perspective-taking, sharing, and turn-taking—combined with parent-focused behaviour management training (BMT), could effectively address externalising behaviours. Kuhn and colleagues (2022) highlight that these approaches yield moderate improvements and outperform alternative methods, presenting a compelling strategy for enhancing program outcomes and reaching more children and families in need.

These comprehensive, systemic parent–child interventions are particularly relevant in the Chilean cultural context, where hierarchical parenting styles often dominate. Such approaches emphasise collaboration and mutual respect, fostering healthier family dynamics that can counteract the behavioural and emotional challenges associated with rigid, hierarchical relationships. Parent-focused BMT, in particular, equips caregivers with tools to use positive reinforcement, emotional attunement, and effective discipline strategies, helping to reduce externalising behaviours while promoting emotional resilience. By addressing both child and parent behaviours, these interventions align with the program’s broader goals and represent a promising strategy for better supporting families through its expanding initiatives.

## Conclusions

This study examined the cascading relationships among emotional (internalising) problems, behavioural (externalising) problems, and language skills in a large general-population sample, followed from early childhood to puberty. The findings provide strong evidence for cross-domain effects, particularly the consistent bidirectional relationship between externalising behaviour and language skills. Gender-stratified analysis revealed (a) a more consistent pattern of externalising behaviour predicting internalising behaviour in females compared to males and (b) bidirectional effects between language skills and externalising behaviour across all time periods studied in males. These findings suggest potential avenues for intervention, highlighting that addressing externalising symptoms and enhancing language skills could yield significant emotional, behavioural, and language benefits during childhood.

## Supplementary Information

Below is the link to the electronic supplementary material.

Supplementary file1 (DOCX 96 KB)

## Data Availability

The datasets produced and analysed in this study are accessible in the Ministerio de Desarrollo social y Familia at: http://observatorio.ministeriodesarrollosocial.gob.cl/elpiprimera-ronda.
