# Supplementary material for: Bidirectional Relationship Between Language Ability and Internalising/Externalising Behaviour from Early to Late Childhood: Findings from a Chilean Cohort
Source: Res Child Adolesc Psychopathol. 2025 Mar 10;53(6):921–35. doi: 10.1007/s10802-025-01302-4 (PMC12137427; doi:10.1007/s10802-025-01302-4)
Supplement: Supplementary file 1 — Supplementary file1 (DOCX 96 KB) [file 10802_2025_1302_MOESM1_ESM.docx]

Supplemental Material

**Supplementary Table 1**

*Table S1 Factor structure of internalizing and externalizing behaviour CBCL scales in the longitudinal sample*

| **Outcome** | **CFI** | **RMSEA** | **SRMR** |
| --- | --- | --- | --- |
| **Wave 1** |  |  |  |
| Internalizing CBCL1 | 0.898 | 0.04 | 0.053 |
| Externalizing CBCL1 | 0.969 | 0.045 | 0.046 |
| **Wave 2**  Internalizing CBCL1 | 0.916 | 0.042 | 0.059 |
| Externalizing CBCL1 | 0.979 | 0.045 | 0.05 |
| Internalizing CBCL2 | 0.942 | 0.039 | 0.055 |
| Externalizing CBCL2 | 0.972 | 0.031 | 0.076 |
| **Wave 3**  Internalizing CBCL2 | 0.949 | 0.035 | 0.05 |
| Externalizing CBCL2 0.958 0.024 0.059 | | | |

**Note.** CFI= Robust Comparative Fit Index, RMSEA= Robust Root Mean Square Error of Approximation, SRMR= Standardized Root Mean Square Residual

Good fit thresholds: CFI ≥ 0.90, RMSEA ≤ 0.05,

SRMR ≤ 0.08.

**Supplementary Table 2**

*Table S2 Correlation estimates for externalizing behaviour, internalizing behaviour and language skills across all covariates*

**Wave 1 Wave 2 Wave 3**

| **Variable** | **EB** | **IB** | **PPVT** | **EB** | **IB** | **PPVT** | **EB** | **IB** | **PPVT** |
| --- | --- | --- | --- | --- | --- | --- | --- | --- | --- |
| Mother age when birth | -0.13*** | -0.15*** | 0.04** | -0.05** | -0.11*** | 0.06*** | 0.02 | -0.05** | 0.06*** |
| Postpartum depression | -0.05** | -0.06*** | -0.03 | -0.05** | -0.05** | -0.03* | -0.04** | -0.03 | -0.03* |
| WAIS vocabulary | -0.19*** | -0.08*** | 0.30*** | -0.13*** | -0.09*** | 0.22*** | -0.08*** | -0.07*** | 0.22*** |
| WAIS numerical | -0.11*** | -0.07*** | 0.25*** | -0.09*** | -0.06*** | 0.14*** | -0.09*** | -0.07*** | 0.14*** |
| Preterm birth | -0.02 | 0.00 | 0.02 | -0.00 | -0.01 | -0.01 | -0.01 | 0.01 | -0.01 |
| Biological father present (1 = yes) | 0.06*** | 0.08*** | -0.03 | 0.05** | 0.09*** | -0.02 | 0.03 | 0.04* | -0.02 |
| Household quintile Wealth | -0.15*** | -0.09*** | 0.21*** | -0.13*** | -0.09*** | 0.18*** | -0.09*** | -0.08*** | 0.18*** |
| Children gender (1 = Female) | 0.03* | -0.08*** | 0.04* | 0.00 | -0.08*** | 0.06*** | -0.06*** | -0.00 | 0.06*** |
| Backward Digit Span Task | -0.11*** | -0.11*** | 0.30*** | -0.09*** | -0.12*** | 0.32*** | -0.06*** | -0.07*** | 0.32*** |
| HOME (affection) | -0.17*** | -0.14*** | 0.17*** | -0.10*** | -0.11*** | 0.08*** | -0.07*** | -0.07*** | -0.00 |
| HOME (does not scold) | -0.08*** | -0.08*** | 0.06*** | -0.13*** | -0.15*** | 0.04*** | 0.01 | -0.00 | 0.06*** |

**Note. ***: *p* < 0.05; ******: *p* < 0.01; *******: *p* < 0.001

EB = Externalising Behaviour; IB = Internalising Behaviour; PPVT = Peabody Picture Vocabulary Test;

HOME (affection) = “Caregiver shows affection (caresses, kisses, cuddles)”; 1 = YES); HOME (does not scold) = “Caregiver does not scold, or derogate or yell” 1 = YES)

**Supplementary Table 3**

|  | **Males** | | **Females** | |  |
| --- | --- | --- | --- | --- | --- |
| **Variable** | **Mean** | **SD** | **Mean** | **SD** | **P-value** |
| Internalizing wave 1 | 59.59 | 9.31 | 60.22 | 9.58 | 0.043 |
| Internalizing wave 2 | 56.11 | 11.42 | 56.19 | 11.63 | 0.833 |
| Internalizing wave 3 | 54.04 | 10.71 | 52.69 | 11.04 | 0.000 |
| Externalizing wave 1 | 60.14 | 10.41 | 58.56 | 10.30 | 0.000 |
| Externalizing wave 2 | 54.56 | 11.74 | 52.64 | 11.37 | 0.000 |
| Externalizing wave 3 | 49.56 | 9.86 | 49.50 | 9.55 | 0.853 |
| Language skills wave 1 | 103.24 | 15.30 | 104.31 | 15.26 | 0.031 |
| Language skills wave 2 | 104.42 | 19.12 | 106.81 | 19.02 | 0.000 |
| Language skills wave 3 | 117.65 | 18.30 | 116.41 | 17.80 | 0.034 |

**Table S3 Gender comparison of internalising, externalising, and language skills across three waves**

*Note* Internalizing = Internalizing Behaviour; Externalizing = Externalizing Behaviour

**Supplementary Table 4**

| **Variables** | **IB w1** | **EB w1** | **PPVT w1** | **IB w2** | **EB w2** | **PPVT w2** | **IB w3** | **EB w3** | **PPVT w3** |
| --- | --- | --- | --- | --- | --- | --- | --- | --- | --- |
| IB w1 | 1.00 | . | . | . | . | . | . | . | . |
| EB w1 | 0.58*** | 1.00 | . | . | . | . | . | . | . |
| PPVT w1 | -0.14*** | -0.14*** | 1.00 | . | . | . | . | . | . |
| IB w2 | 0.33*** | 0.25*** | -0.11*** | 1.00 | . | . | . | . | . |
| EB w2 | 0.25*** | 0.42*** | -0.12*** | 0.68*** | 1.00 | . | . | . | . |
| PPVT w3 | -0.11*** | -0.12*** | 0.42*** | -0.06*** | -0.08*** | 1.00 | . | . | . |
| IB w3 | 0.26*** | 0.20*** | -0.05*** | 0.26*** | 0.22*** | -0.05** | 1.00 | . | . |
| EB w3 | 0.18*** | 0.27*** | -0.04* | 0.19*** | 0.30*** | -0.06*** | 0.66*** | 1.00 | . |
| PPVT w3 | -0.13*** | -0.10*** | 0.37*** | -0.11*** | -0.14*** | 0.40*** | -0.03* | -0.07*** | 1.00 |

**Table S4 Cross-sectional correlations of internalizing, externalizing and language skills**

*Note* IB = Internalizing Behaviour; EB = Externalizing Behaviour; PPVT = Language skills

w1 = Wave 1; w2 = Wave 2; w3 = Wave 3

**Supplementary Table 5**

*Table S5 Regression coefficients for all paths in the Cross Lagged Panel Model, with and without adjustment for covariates*

| **Path Description Path Coefficient (no covariates) Path Coefficient (covariates)** | | |
| --- | --- | --- |
| T1 Externalizing *→* T2 Externalizing | 0.41*** | 0.39*** |
| T2 Externalizing *→* T3 Externalizing | 0.34*** | 0.33*** |
| T1 Internalizing *→* T2 Internalizing | 0.31*** | 0.28*** |
| T2 Internalizing *→* T3 Internalizing | 0.22*** | 0.20*** |
| T1 PPVT *→* T2 PPVT | 0.41*** | 0.35*** |
| T2 PPVT *→* T3 PPVT | 0.40*** | 0.36*** |
| T1 Externalizing *→* T2 Internalizing | 0.05** | 0.05** |
| T2 Externalizing *→* T3 Internalizing | 0.07*** | 0.06** |
| T1 Externalizing *→* T2 PPVT | -0.05*** | -0.04** |
| T2 Externalizing *→* T3 PPVT | -0.07*** | -0.07*** |
| T1 Internalizing *→* T2 Externalizing | 0.03 | 0.00 |
| T2 Internalizing *→* T3 Externalizing | -0.04 | -0.06** |
| T1 Internalizing *→* T2 PPVT | -0.04** | 0.00 |
| T2 Internalizing *→* T3 PPVT | -0.05** | -0.01 |
| T1 PPVT *→* T2 Externalizing | -0.05*** | -0.03* |
| T2 PPVT *→* T3 Externalizing | -0.04** | -0.02 |
| T1 PPVT *→* T2 Internalizing | -0.06*** | -0.02 |
| T2 PPVT *→* T3 Internalizing | -0.04* | -0.01 |

**Note.** Externalizing = Externalizing Behaviour; Internalizing = Internalizing Behaviour; PPVT = Peabody Picture

Vocabulary Test

**Supplementary Table 6**

*Table S6 Regression coefficients for all paths in the Random Intercept Cross-Lagged Panel Model, with adjustment for covariates and their 95% confidence intervals (full sample)*

| **Path Description Path Coefficients (covariates) 95 % CI** | | |
| --- | --- | --- |
| T1 Externalising *→* T2 Externalising | 0.33*** | [0.29, 0.37] |
| T1 Internalising → T3 Externalising | -0.03* | [-0.07, -0.00] |
| T1 PPVT *→* T2 Externalizing | -0.05* | [-0.09, -0.01] |
| T1 Externalizing *→* T2 Internalizing | 0.02 | [-0.02, 0.06] |
| T1 Internalizing *→* T2 Internalizing | 0.16*** | [0.12, 0.21] |
| T1 PPVT *→* T2 Internalizing | -0.02 | [-0.06, 0.28] |
| T1 Externalizing *→* T2 PPVT | -0.07*** | [-0.10, -0.03] |
| T2 Internalising *→* T2 PPVT | -0.04 | [-0.07, 0.01] |
| T1 PPVT *→* T2 PPVT | 0.09** | [0.03, 0.14] |
| T2 Externalizing *→* T3 Externalising | 0.15*** | [0.08, 0.22] |
| T2 Internalising → T3 Externalizing | -0.07* | [-0.13, -0.01] |
| T2 PPVT *→* T3 Externalizing | -0.04 | [-0.09, 0.01] |
| T2 Externalising *→* T3 Internalising | 0.09*** | [0.04, 0.14] |
| T2 Internalising. *→* T3 Internalising | 0.04 | [-0.01, 0.10] |
| T2 PPVT *→* T3 Internalizing | -0.05* | [-0.09, -0.01] |
| T2 Externalising *→* T3 PPVT | -0.05* | [-0.09, -0.01] |
| T1 Internalising *→* T2 PPVT | -0.08*** | [-0.13, -0.04] |
| T2 PPVT *→* T3 PPVT | 0.14*** | [0.09, 0.18] |

**Note.** Externalizing = Externalizing Behaviour; Internalizing = Internalizing Behaviour; PPVT = Peabody Picture Vocabulary Test

**Supplementary Table 7**

*Table S7 Regression coefficients for all paths in the Random Intercept Cross-Lagged Panel Model, with adjustment for covariates and their 95% confidence intervals (males)*

| **Path Description Path Coefficients (covariates) 95 % CI** | | |
| --- | --- | --- |
| T1 Externalising *→* T2 Externalising | 0.31*** | [0.24, 0.38] |
| T1 Internalising → T3 Externalising | -0.05 | [-0.11, 0.02] |
| T1 PPVT *→* T2 Externalizing | -0.05 | [-0.11, 0.00] |
| T1 Externalizing *→* T2 Internalizing | 0.05 | [-0.02, 0.12] |
| T1 Internalizing *→* T2 Internalizing | 0.11** | [0.03, 0.18] |
| T1 PPVT *→* T2 Internalizing | -0.04 | [-0.10, 0.02] |
| T1 Externalizing *→* T2 PPVT | -0.11** | [-0.17, -0.04] |
| T2 Internalising → T2 PPVT | -0.00 | [-0.07, 0.07] |
| T1 PPVT *→* T2 PPVT | 0.03 | [-0.05, 0.11] |
| T2 Externalizing *→* T3 Externalising | 0.18*** | [0.08, 0.28] |
| T2 Internalising → T3 Externalizing | -0.06 | [-0.14, 0.03] |
| T2 PPVT *→* T3 Externalizing | -0.08** | [-0.15, -0.02] |
| T2 Externalising *→* T3 Internalising | 0.08 | [-0.00, 0.17] |
| T2 Internalising. *→* T3 Internalising | 0.03 | [-0.06, 0.11] |
| T2 PPVT *→* T3 Internalizing | -0.01 | [-0.07, 0.04] |
| T2 Externalising *→* T3 PPVT | -0.13** | [-0.21, -0.04] |
| T1 Internalising *→* T2 PPVT | 0.01 | [-0.07, 0.09] |
| T2 PPVT *→* T3 PPVT | 0.11** | [0.04, 0.17] |

**Note.** Externalizing = Externalizing Behaviour; Internalizing = Internalizing Behaviour; PPVT = Peabody Picture Vocabulary Test

**Supplementary Table 8**

*Table S8 Regression coefficients for all paths in the Random Intercept Cross-Lagged Panel Model, with adjustment for covariates and their 95% confidence intervals (females)*

| **Path Description Path Coefficients (covariates) 95 % CI** | | |
| --- | --- | --- |
| T1 Externalising *→* T2 Externalising | 0.26*** | [0.19, 0.32] |
| T1 Internalising → T3 Externalising | -0.01 | [-0.08, 0.05] |
| T1 PPVT *→* T2 Externalizing | -0.04 | [-0.10, 0.03] |
| T1 Externalizing *→* T2 Internalizing | 0.07* | [0.01, 0.13] |
| T1 Internalizing *→* T2 Internalizing | 0.05 | [-0.03, 0.13] |
| T1 PPVT *→* T2 Internalizing | 0.00 | [-0.06, 0.07] |
| T1 Externalizing *→* T2 PPVT | -0.10*** | [-0.16, -0.04] |
| T2 Internalising → T2 PPVT | 0.04 | [-0.03, 0.11] |
| T1 PPVT *→* T2 PPVT | 0.15*** | [0.07, 0.22] |
| T2 Externalizing *→* T3 Externalising | 0.13* | [0.03, 0.24] |
| T2 Internalising → T3 Externalizing | -0.06 | [-0.14, 0.03] |
| T2 PPVT *→* T3 Externalizing | -0.01 | [-0.08, 0.05] |
| T2 Externalising *→* T3 Internalising | 0.13** | [0.04, 0.22] |
| T2 Internalising. *→* T3 Internalising | -0.04 | [-0.13, 0.05] |
| T2 PPVT *→* T3 Internalizing | 0.00 | [-0.06, 0.06] |
| T2 Externalising *→* T3 PPVT | -0.14*** | [-0.22, -0.06] |
| T1 Internalising *→* T2 PPVT | 0.05 | [-0.03, 0.13] |
| T2 PPVT *→* T3 PPVT | 0.18*** | [0.11, 0.25] |

**Note.** Externalizing = Externalizing Behaviour; Internalizing = Internalizing Behaviour; PPVT = Peabody Picture Vocabulary Test

**Supplementary Table 9**

*Table S9: Regression coefficients for all paths in the Random Intercept Cross-Lagged Panel Model for the sensitivity analysis, with adjustments for covariates and their 95% confidence intervals (full sample).*

| **Path Description Path Coefficients (covariates) 95 % CI** | | |
| --- | --- | --- |
| T1 Externalising *→* T2 Externalising | 0.33 *** | [0.28, 0.39] |
| T1 Internalising → T3 Externalising | -0.03 * | [-0.24, 0.03] |
| T1 PPVT *→* T2 Externalizing | -0.05 ** | [-0.03, -0.00] |
| T1 Externalizing *→* T2 Internalizing | 0.02 | [-0.01, 0.03] |
| T1 Internalizing *→* T2 Internalizing | 0.16 *** | [0.10, 0.23] |
| T1 PPVT *→* T2 Internalizing | -0.02 | [-0.01, 0.00] |
| T1 Externalizing *→* T2 PPVT | -0.07 *** | [-0.60, -0.13] |
| T2 Internalising → T2 PPVT | -0.04 * | [-1.47, -0.02] |
| T1 PPVT *→* T2 PPVT | 0.09 ** | [-0.07, 0.15] |
| T2 Externalizing *→* T3 Externalising | 0.15 *** | [0.07, 0.19] |
| T2 Internalising → T3 Externalizing | -0.07 * | [-0.24, 0.01] |
| T2 PPVT *→* T3 Externalizing | -0.04 | [-0.02, 0.00] |
| T2 Externalising *→* T3 Internalising | 0.09 *** | [0.01, 0.06] |
| T2 Internalising. *→* T3 Internalising | 0.04 | [0.01, 0.11] |
| T2 PPVT *→* T3 Internalizing | -0.05 * | [-0.02, 0.10] |
| T2 Externalising *→* T3 PPVT | -0.05 * | [-0.51, 0.04] |
| T1 Internalising *→* T2 PPVT | -0.08 *** | [-2.06, -0.46] |
| T2 PPVT *→* T3 PPVT | 0.14 *** | [0.04, 0.17] |

**Note.** Externalizing = Externalizing Behaviour; Internalizing = Internalizing Behaviour; PPVT = Peabody Picture Vocabulary Test

**Supplementary Table 10**

*Table S10: Regression coefficients for all paths in the Random Intercept Cross-Lagged Panel Model for the sensitivity analysis, with adjustments for covariates and their 95% confidence intervals (males).*

| **Path Description Path Coefficients (covariates) 95 % CI** | | |
| --- | --- | --- |
| T1 Externalising *→* T2 Externalising | 0.34*** | [0.28, 0.39] |
| T1 Internalising → T3 Externalising | -0.04 | [-0.24, 0.03] |
| T1 PPVT *→* T2 Externalizing | -0.05* | [-0.03, -0.00] |
| T1 Externalizing *→* T2 Internalizing | 0.03 | [-0.01, 0.03] |
| T1 Internalizing *→* T2 Internalizing | 0.16*** | [0.10, 0.23] |
| T1 PPVT *→* T2 Internalizing | -0.03 | [-0.01, 0.00] |
| T1 Externalizing *→* T2 PPVT | -0.08** | [-0.60, -0.13] |
| T2 Internalising → T2 PPVT | -0.05 | [-1.47, -0.02] |
| T1 PPVT *→* T2 PPVT | 0.14*** | [-0.07, 0.15] |
| T2 Externalizing *→* T3 Externalising | 0.07 | [0.07, 0.19] |
| T2 Internalising → T3 Externalizing | -0.06 | [-0.24, 0.01] |
| T2 PPVT *→* T3 Externalizing | -0.07* | [-0.02, 0.00] |
| T2 Externalising *→* T3 Internalising | 0.11*** | [0.01, 0.06] |
| T2 Internalising. *→* T3 Internalising | 0.05 | [0.01, 0.11] |
| T2 PPVT *→* T3 Internalizing | -0.08* | [-0.02, 0.10] |
| T2 Externalising *→* T3 PPVT | -0.05 | [-0.51, 0.04] |
| T1 Internalising *→* T2 PPVT | -0.10** | [-2.06, -0.46] |
| T2 PPVT *→* T3 PPVT | 0.11*** | [0.04, 0.17] |

**Note.** Externalizing = Externalizing Behaviour; Internalizing = Internalizing Behaviour; PPVT = Peabody Picture Vocabulary Test

**Supplementary Table 11**

*Table S10: Regression coefficients for all paths in the Random Intercept Cross-Lagged Panel Model for the sensitivity analysis, with adjustments for covariates and their 95% confidence intervals (females).*

| **Path Description Path Coefficients (covariates) 95 % CI** | | |
| --- | --- | --- |
| T1 Externalising *→* T2 Externalising | 0.31*** | [0.23, 0.34] |
| T1 Internalising → T3 Externalising | -0.03 | [-0.19, 0.05] |
| T1 PPVT *→* T2 Externalizing | -0.04 | [-0.02, 0.00] |
| T1 Externalizing *→* T2 Internalizing | 0.02 | [-0.01, 0.02] |
| T1 Internalizing *→* T2 Internalizing | 0.17*** | [0.10, 0.23] |
| T1 PPVT *→* T2 Internalizing | -0.01 | [-0.01, 0.01] |
| T1 Externalizing *→* T2 PPVT | -0.05* | [-0.47, -0.01] |
| T2 Internalising → T2 PPVT | -0.02 | [-1.02, 0.42] |
| T1 PPVT *→* T2 PPVT | 0.14*** | [0.08, 0.28] |
| T2 Externalizing *→* T3 Externalising | 0.07 | [-0.03, 0.11] |
| T2 Internalising → T3 Externalizing | -0.06 | [-0.23, 0.05] |
| T2 PPVT *→* T3 Externalizing | -0.01 | [-0.01, 0.01] |
| T2 Externalising *→* T3 Internalising | 0.06 | [-0.01, 0.04] |
| T2 Internalising. *→* T3 Internalising | 0.03 | [-0.04, 0.10] |
| T2 PPVT *→* T3 Internalizing | -0.08 | [-0.01, 0.00] |
| T2 Externalising *→* T3 PPVT | -0.06* | [-0.60, 0.02] |
| T1 Internalising *→* T2 PPVT | -0.10** | [-1.65, -0.05] |
| T2 PPVT *→* T3 PPVT | 0.18*** | [0.10, 0.23] |

**Note.** Externalizing = Externalizing Behaviour; Internalizing = Internalizing Behaviour; PPVT = Peabody Picture Vocabulary Test
